# A directional wave measurement attack against the Kish key distribution system

**DOI:** 10.1038/srep06461

**Published:** 2014-09-24

**Authors:** Lachlan J. Gunn, Andrew Allison, Derek Abbott

**Affiliations:** 1 School of Electrical and Electronic Engineering, The University of Adelaide, SA 5005, Australia

## Abstract

The Kish key distribution system has been proposed as a classical alternative to quantum key distribution. The idealized Kish scheme elegantly promises secure key distribution by exploiting thermal noise in a transmission line. However, we demonstrate that it is vulnerable to nonidealities in its components, such as the finite resistance of the transmission line connecting its endpoints. We introduce a novel attack against this nonideality using directional wave measurements, and experimentally demonstrate its efficacy.

As early as the 1940s, the idea of exploiting classical noise for secure communication has been considered^1^. However, these early scrambling systems would resist cryptanalysis for hours^1^, rather than years as we have come to expect, and so became a forgotten footnote in cryptographic history.

The idealized Kish key distribution (KKD) system, based on Kirchhoff's laws and Johnson noise (KLJN)^2^ has been proposed as a classical alternative to quantum key distribution (QKD)^3^. Eschewing expensive and environmentally-sensitive optics, practical KKD can be implemented economically in a wider variety of systems than QKD. Such information-theoretic systems have been of great interest since the development of Shor's algorithm^4^, which, if successfully implemented on a significant scale, will potentially break most key-distribution schemes in use today.

The KKD system is claimed^2^ to derive unconditional security from the second law of thermodynamics—the idea being that net power cannot flow from one resistor to the other under equilibrium.

An idealised KKD system is shown in Figure 1. Alice and Bob each apply a noise signal to a line through a series resistor. The voltage on the line is unchanged if the terminals of Alice and Bob are swapped; if the mean-square voltages applied by Alice and Bob are proportional to *R_a_* and *R_b_* respectively then no average power flows through the line, and in the ideal case an eavesdropper, Eve, cannot determine which end has which resistance^2^,^5^. If Alice and Bob randomly choose their resistances—resulting in corresponding noise amplitudes—to be either *R_h_* or *R_l_*, three possibilities avail themselves: both choose *R_h_*, both choose *R_l_*, or one chooses *R_h_* and the other chooses *R_l_*. In this third case, Alice knows the value of her own resistor, and so can deduce Bob's resistor via noise spectral analysis, and vice-versa. However, an eavesdropper lacks this knowledge, and so in the ideal case Alice and Bob have secretly shared one bit of information. This then forms the basis for Alice and Bob secretly sharing random numbers that can be exploited as secure cryptographic keys.

It has been claimed^6^ that transmission line theory does not apply to the KKD system when operated at frequencies below *f_c_* = *ν*/(2*L*), where *L* is the transmission line length and *ν* the signal propagation velocity, because wave modes do not propagate below this cutoff frequency. We demonstrate that this is not the case by constructing a directional wave measurement device that is then used for a successful finite-resistance attack against the system. The position that frequencies below *f_c_* do actually propagate is also supported by the fact that, at low frequencies, a coaxial cable is known to only support TEM modes—these modes are known to have no low frequency cutoff^7^. An exception occurs when the two ends of the line are held at equal potential; only standing waves possessing a frequency that is an integer multiple of *ν*/(2*L*) can fulfill these boundary conditions^8^. However, the the KKD system differs in allowing arbitrary potentials to appear at the ends of the line, and so supports propagating waves.

Several attacks against the KKD system exist, however none thus far have been shown experimentally to substantially reduce the security of the system^9^.

The first attacks, proposed by Scheuer and Yariv^10^, rely upon imperfections in the line connecting the two terminals; the first exploits transients generated by the resistor-switching operation, while the second exploits the line's finite resistance. The former is foiled by the addition of low-pass filters to the terminals^11^, while the latter was shown to leak fewer than 1% of bits^9^,^11^ in a practical system.

An attack by Hao^12^,^13^ instead focuses upon imperfections of the terminals; inaccuracies in the noise temperatures of Alice and Bob create an information leak. However, it was demonstrated^9^,^13^ that noise can be digitally generated with a sufficiently accurate effective noise temperature to prevent this attack from being useful in practice.

A theoretical argument has been made by Bennett and Riedel^14^ that no purely classical electromagnetic system can be unconditionally secure due to the structure of Maxwell's equations. It is argued that the upper bound on secrecy rate by Maurer^15^ must be zero because of the locally-causal nature of classical electromagnetics, and so an eavesdropper can perfectly reconstruct the key with the aid of a directional coupler. Kish, et al.^16^ responded that a nonzero secrecy rate is unnecessary in practice, provided it can be achieved in the ideal limit.

## Results

### Circuit analysis

**Figure 1:**
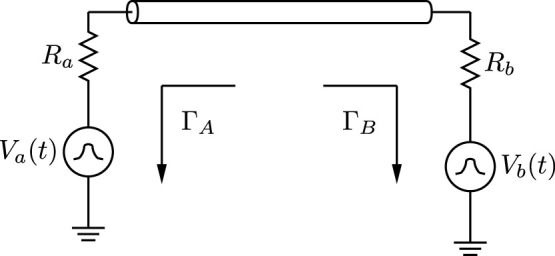
The idealized KKD system. Practical systems include low-pass filters and instrumentation that do not affect the steady-state signal. The mean-squared voltages 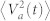 and 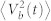 are proportional to the resistances *R_a_* and *R_b_* respectively. Note that in a practical system, artificial noise sources are used, and thus the equivalent noise temperature in our experiment is 3.62 × 10^15^ K. This is equivalent to 1 V RMS of voltage noise with a 1 kΩ resistor over a bandwidth of 5 kHz. We perform our analysis in terms of the reflection coefficients Γ*_A_* and Γ*_B_*.

We begin our attack by analyzing the system in Figure 1 to determine the forward- and reverse-travelling waves through the transmission line. Let us denote the equivalent noise voltages of Alice and Bob *V_a_*(*t*) and *V_b_*(*t*) respectively, and the waves injected onto the line 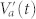 and 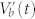. These are related by 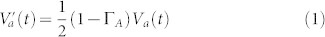

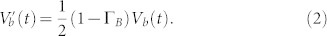


Noting that the mean-squared thermal noise voltage is 〈*V*^2^〉 = 4*kTBR*, we find that 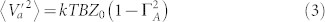

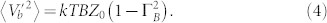


As the transmission line in the KKD system is short—and so the forward- and reverse-travelling waves are equal throughout the line except for a loss factor *α*—we may write the left- and right-travelling waves at Bob's and Alice's ends of the line respectively as 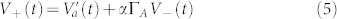

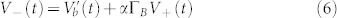
and so 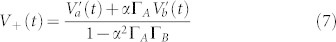

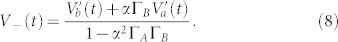


We may write this in matrix form **v***_d_*(*t*) = *A***v***_i_*(*t*) and so find the covariance matrix 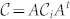 of the directional components: 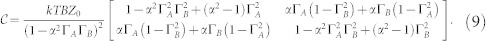
When the line is lossless and so *α* = 1, Eqn. 9 is invariant under permutation of Γ*_A_* and Γ*_B_*, and so the covariance matrix provides no information on the choice of resistors. However, when *α* < 1 this property fails to hold, allowing the choices of Γ*_A_* and Γ*_B_* to be determined from the distribution of (*v*_+_, *v*_−_); this allows an eavesdropper to attack the system by performing a statistical test between the two possible covariance matrices. Note that we need not measure the generator voltages themselves—which an eavesdropper cannot directly access—but merely the waves travelling in each direction.

### Statistical processing

We have derived a statistical representation of the noise that travels along the transmission line; while we might measure the power travelling in each direction in order to determine the resistor configuration, the distributions to be distinguished are very similar, resulting in a relatively large bit-error rate (BER) as was shown in^11^. However, comparison of the variances of *v*_+_ and *v*_−_ is suboptimal. We derive an improved test using Bayesian methods and demonstrate that the two cases can be far more easily distinguished than with a direct difference-of-mean-squares test of Scheuer and Yariv^10^.

Knowing the covariance matrices of *v*_+_(*t*) and *v*_−_(*t*) for each hypothesis, we may use Bayes' theorem^17^ to determine the probability of each configuration. Let *C* = 0 and *C* = 1 refer to the events that (*R_a_*,*R_b_*) = (*R_h_*,*R_l_*) and vice-versa, respectively. Then, 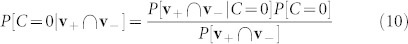

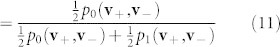

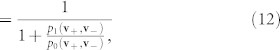
where *p*_0_(·,·) and *p*_1_ (·,·) are the *multivariate* Gaussian PDFs for the measurements from each respective configuration.

The most probable state, then, is given by the maximum-likelihood estimator^17^

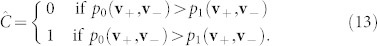
The comparison is more conveniently made in terms of the log-likelihood, which for the *n*-variate zero-mean Gaussian distribution with covariance matrix Σ is given by^18^

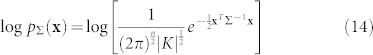

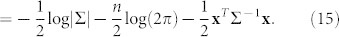


Noting that Σ is positive-definite, we may therefore write it in terms of its Cholesky decomposition Σ = *KK^T^*, and so 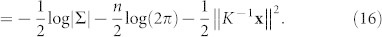
Only the final term depends upon the data, and there only through the total power of a group of signals *K*^−1^**x** formed by linear combinations of the measured waves.

**Figure 2:**
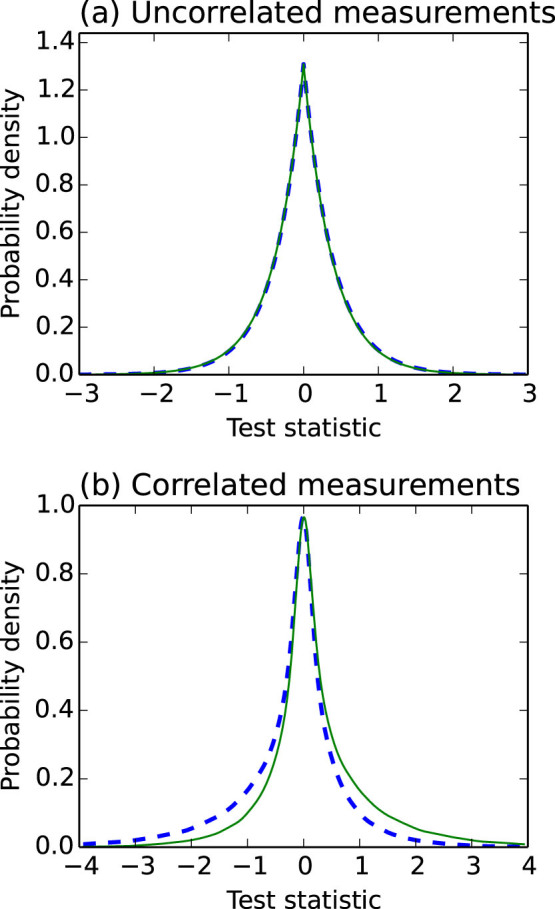
Log likelihood-ratio test statistics for each permutation of resistors in Eqn. 9, as in Eqn. 16 with scaling-factors omitted. The dashed lines correspond to the case where (*R_a_*, *R_b_*) = (*R_l_*, *R_h_*), and the solid lines to (*R_a_*, *R_b_*) = (*R_h_*, *R_l_*). Parameters are *R_l_* = 1 kΩ, *R_h_* = 10 kΩ, *Z*_0_ = 50 Ω, and *α* = −0.1 dB. In (a) the covariances are set to zero, and so Eqn. 13 reduces to a simple power comparison. The distributions are almost indistinguishable. In (b), the measurement variables are drawn from a correlated bivariate distribution having the same marginal variances, and are far more distinguishable. In either case, as losses increase and so the variances of the measurements and transformed measurements respectively differ more greatly, the two distributions, which mirror each other about zero, become increasingly assymmetric and so far more distinguishable.

It should be noted that this estimator differs substantially from that proposed by Scheuer and Yariv^10^, which makes a simple comparison of variances. The measured variables in our case are collected simultaneously and so exhibit the heavy correlations of Eqn. 9. With these correlations, the likelihood-ratio test provides far better performance than the difference in the variances of the marginal distributions would suggest. However, if the voltage and current measurements are considered separately, as in^9^,^11^ where only the marginal distributions of each measurement are computed, these correlations vanish and so the estimator described in Eqns. 13 and 16 has substantially less power. The distribution of test statistics is shown in Figure 2 for a loss of 0.1 dB. The presence of correlation causes the distributions of test statistics to differ substantially, where otherwise they would be almost indistinguishable.

**Figure 3:**
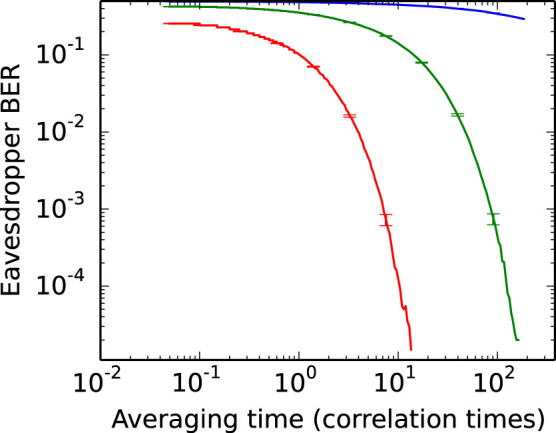
Simulated eavesdropper bit-error-rate as a function of averaging time, for line attenuations of 0.01, 0.1, and 1.0 decibels respectively from top to bottom. The link parameters are *R_L_* = 1 kΩ, *R_H_* = 1 kΩ, *Z*_0_ = 50 Ω. Note that the averaging time is expressed in multiples of 200 μs. This is the correlation time (i.e. reciprocal of the system bandwidth) so that the results are bandwidth independent. Transmission lines with greater loss are more susceptible to attack, with substantial attenuations providing little protection. The error rates are estimated from a sample size of 10^5^, with 2*σ* error bars shown.

The results of simulation for various values of loss are shown in Figure 3. A pair of white noise processes are generated, Fourier-transformed, and the undesirable frequency components removed. They are combined according to Eqn. 8 to produce the voltage waves, and the maximum-likelihood estimator is used to determine the resistor configurations. This demonstrates that our estimator can differentiate the two distributions without the unreasonably large sample sizes that were previously thought necessary^11^.

### Experimental results

**Figure 4:**
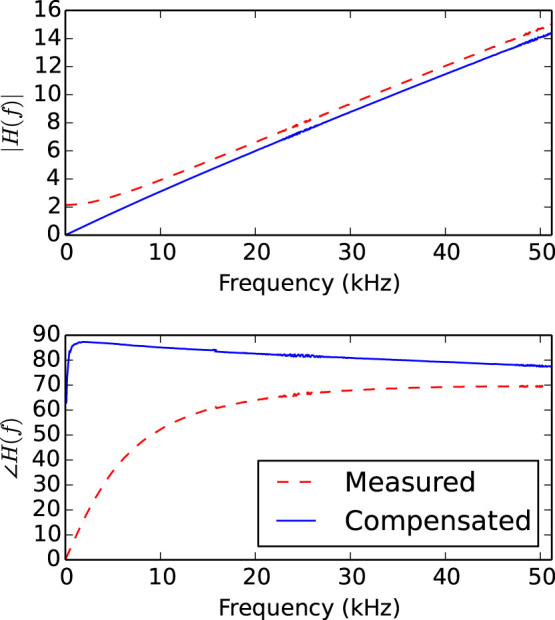
Measured frequency response of the *∂v*/*∂x* estimation circuit in Figure 6. The derivative increases linearly with frequency, as would be expected from the d'Alembert solution to the wave equation. The response *H*(0) at DC is subtracted in order to remove the effect of wire resistance, yielding the ‘compensated’ curves above. After this correction we see 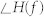 approximating the expected +90° constant phase response, slightly drooping due to the limited frequency response of the system.

Having demonstrated our attack in simulation, we proceed to experimental validation of the model. The estimation of *∂v*/*∂x* is key to the operation of the device, however the circuit synthesis is dependent upon a wave-based analysis of the system. We therefore measure experimentally the frequency response of the electronically-estimated *∂v*/*∂x*, shown in Figure 4, with a wave travelling in a single direction in order to verify that our analysis is appropriate.

We expect to see a magnitude response linear in frequency and a constant +90° phase response. This agrees with the experimental results shown in Figure 4, validating our analysis, and demonstrates that the signal through a short transmission line indeed propagates as a wave, in contradiction to the theoretical claims of Kish and Horvath^6^.

**Figure 5:**
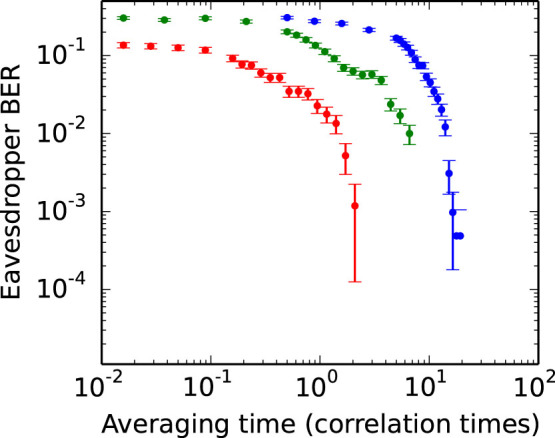
Measured eavesdropper bit-error-rate as a function of averaging time and line attenuation. The line is approximately 2 m in length and has a loss of less than 0.1 dB. From top to bottom, 0 dB, 0.1 dB, and 1 dB of additional attenuation provided by inserting an in-line attenuator at one end of the line.

We measure the voltage components in each direction and compute the log-likelihoods (16). Their differences are thresholded to compute (13); the bit error rates for various averaging times and line parameters are shown in Figure 5. Even modest losses, below 0.1 dB, allow more than 99.9% of bits to be determined correctly in less than 20 correlation times, showing that the technique simulated in Figure 3 can be applied in practice.

### Proposed countermeasures and alternative explanations

Several countermeasures to and alternative explanations of this attack have been proposed in response to a preprint of this paper; we take a moment to discuss each of these.

#### Arguments against the transmission-line model of the KKD system

It is argued^16^,^19^ on several grounds that the wave-based model that we have used is inaccurate. It is first claimed that the wave equation on a finite domain does not admit sinusoidal solutions other than of frequencies *f_k_* = *kν*/2*L*, where *ν* is the propagation velocity and *L* the length of the transmission line. However, this quantisation effect is induced by boundary conditions of the form *v*(0) = *v*(*L*); in the KKD system, resistive terminations allow arbitrary potentials to appear at the two ends of the line and so this does not occur. We also note that these spatial frequencies do not directly correspond to temporal frequencies in the injected signals, but are instead indicative of the spatial spectrum of the periodic extension of the voltage distribution along the line.

It is next claimed by Chen et al^19^ that the signals within the KKD system cannot be waves because their energy does not exchange between electric and magnetic fields. However this will always be the case. Consider an infinitely long coaxial cable driven by a sinusoidal source *V*_0_(*t*). It is shown by Chen et al^19^ that the relationship between the instantaneous voltages and currents in a small initial segment of the line will cause the energy to be evenly split between electric and magnetic fields. As we are considering an infinitely long coaxial cable, the voltages and currents contain no reflected components, and so will be given by


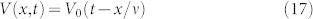

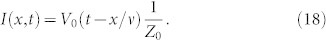


The distribution of energy between electric and magnetic fields therefore does not change as the signal propagates along the transmission line. The voltages and currents are known^20^ to satisfy the wave equation, and yet they do not exchange energy in the manner suggested by Chen et al^19^.

It is further claimed that a lack of discretisation of frequencies disagrees with the calculations of Planck and would invalidate Planck's Law. However, it is incorrectly claimed by Chen et al^19^ that Planck's Law is derived for radiation inside a black-sided box; in fact, the box analysed by Planck^21^ is perfectly conductive. It is these perfectly conductive edges that induce quantisation of the spatial frequencies^21^. In simple terms, recall that Planck's formulation solves the ultraviolet catastrophe by introducing an *upper* frequency cut-off via quantisation. An attempt by Chen et al^19^ to use this analogy to argue for a *lower* frequency cut-off in a coax line is therefore not valid and appears to have the situation inverted.

Another argument^19^ has been made against the presence of waves using the equipartition theorem. It is claimed that the equipartition theorem requires each wave mode of the transmission line to possess an energy of 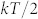, and that for a line in thermal equilibrium with the generators, the power on the line is insufficient to excite even a single wave mode. However, the non-idealized KKD system is not a thermodynamically closed system, but uses artificial noise sources and has resistive terminations. These terminations dissipate power into the environment, and the noise sources must be supplied with external power; the KKD system therefore is not in thermal equilibrium and the equipartition theorem does not apply.

It is also claimed by Chen et al^19^, based on a lumped-model analysis, that the phase velocity of the propagating signal is dependent upon the line terminations, invalidating the use of the d'Alembert solution to the wave equation. However, this analysis conflates phase and propagation velocities, and similar results—identical except for the addition of propagation time—can be derived from a wave-based analysis (see Supplementary Note S1). We note also that, contrary to the claims of Chen et al^19^, for *guided* modes, superluminal phase velocities do not violate special relativity as they do not imply superluminal wave signal propagation^22^,^23^.

Contrary to the implication of Chen et al^19^, there is no definitive definition of a wave in the literature. Even attempting to define a wave as a solution of the wave equation is overly restrictive, as waves in dispersive media do not strictly satisfy the standard wave equation^23^. Thus physics texts (e.g.^24^) define a wave in the broadest possible terms as a transfer of energy from one state to another with a finite velocity. A wave does not even need to be periodic—for example, it can be overdamped or even chirped. It appears that, in each argument Chen et al^19^, preselects its own *ad hoc* definition of what a wave is in order to arrive at a non-standard viewpoint.

#### Experimental critique

**Figure 6:**
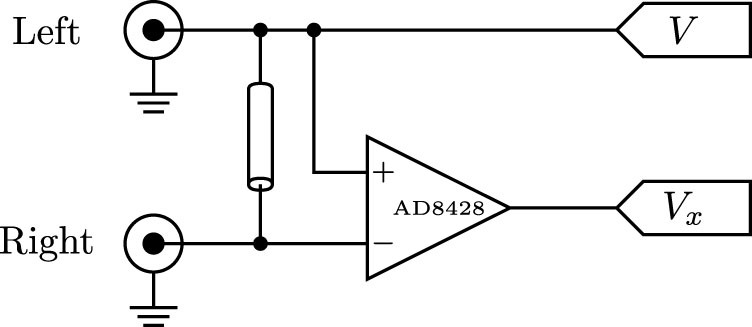
The analog frontend of the directional wave measurement device. Buffering, offset, gain control, and clamping are not shown. An instrumentation amplifier is used to measure the voltage across a 1.5 m length of coaxial cable, providing an estimate of *∂v*/*∂x*. After offset and gain adjustments, the signals are simultaneously sampled by the 12-bit ADCs of an STM32F407 microcontroller.

**Figure 7:**
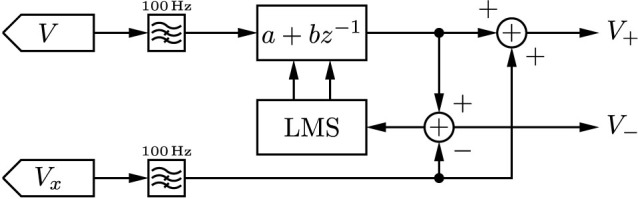
The digital signal processing of the directional wave measurement device, implemented on an STM32F407 microcontroller. A least-mean-squares filter is used at startup to determine the necessary filter coefficients; a signal is applied to one port while the other is connected to a terminator, and the filter coefficients adjusted to force *V*_−_ = 0. Filter updates are disabled once the apparent reflection coefficient becomes sufficiently small.

It was suggested by Chen et al^25^ that mains interference or DC offsets, might be responsible for our results, as they would produce an apparent DC offset during each measurement. Note that DC offsets are removed by high-pass filtering after digitisation, as shown in Figure 7, and 50 Hz interference is suppressed as well. The delay line is shielded by the coaxial braid, and is wound in a non-inductive bifilar configuration^26^ in order to further reduce mains pickup. The magnitude of the 50 Hz interference measured on the *V_x_* channel—see Figure 6—is 15 mV RMS after amplification, and remains constant whether or not a complete circuit exists through the two resistors to ground, thus suggesting this effect to be insignificant on that channel. Interference picked up by the *V* channel—the quantity considered by Chen et al^25^—increases with the establishment of a current loop, but at 40 μV RMS this is more than 85 dB below the generator signal, and so insignificant in the short time over which we average.

It is suggested^19^ that our apparatus might have non-Gaussian signals present, and that this known vulnerability might be responsible for our results. However, our method uses only second-order statistics, and so does not depend upon the distributions of the signals, but merely their variances and correlations, which can be trivially computed as above.

#### Proposed countermeasures

A countermeasure to finite-resistance attacks has been proposed by Kish and Granqvist^27^. They propose to boost the noise temperature of one source in order to compensate for the extra resistance of the cable.

While their analysis considers only lumped models, our analysis shows that this type of countermeasure is effective against our attack, requiring the temperatures to be varied according to 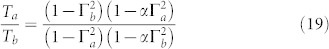
under our model. This allows our attack in its current form to be defeated if *α* can be accurately measured by the two parties. However, it remains for future work to determine if this can be implemented in a secure manner, as the measurement protocol for *α* remains unspecified.

## Discussion

The technique above exploits imperfections in the KKD implementation; while it might be theoretically possible to counter this attack by reduction of losses as proposed by Kish^11^, the reduction of losses substantially below 0.1 dB ensures that this will be infeasible for all but the shortest or slowest of links.

This raises the question of why our attack should succeed where existing finite-resistance attacks have failed. The attack of Scheuer and Yariv^10^ considered only the variances of the measured variables. Our attack exploits the large correlation between waves in each direction; the estimator used above partially removes this common signal, increasing the ability to distinguish between the two cases statistically.

We have demonstrated an attack against the KKD key distribution system that exploits losses within the connecting transmission line. The attack has been shown experimentally to correctly determine more than 99.9% of bits transmitted over a 2 m transmission line within 20 correlation times. As this attack requires that losses be reduced to a fraction of a decibel in order to maintain a meaningful level of security, modifications to the system, such as proposed by Kish and Granqvist^27^, will be necessary in order to produce a secure link of any significant length and bitrate.

## Methods

A directional coupler separates forward- and reverse-travelling waves on a transmission line^20^. We have constructed a similar device using differential measurements across a delay line, shown in Figure 6.

Consider the d'Alembert solution^7^ to the wave equation in a medium with propagation velocity *ν*, 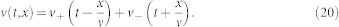
The forward-travelling component *v*_+_(*τ*) differs from the reverse-travelling component *v*_−_(*τ*) in the sign of its spatial argument. We use this to our advantage by computing the linear combinations 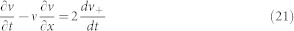

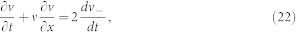
yielding the forward- and reverse-travelling waves as we desire. All that remains, then, is to determine *∂v*/*∂t* and *∂v*/*∂x*.

The time derivative *∂v*/*∂t* may be determined digitally from sampled values of *v*(*t*). The spatial derivative is approximated as being proportional to the voltage across a short delay line, shown in Figure 6.

After digitisation, we high-pass filter the signals *V* and *V_x_* in order to remove any DC offsets or mains interference. The signals are then combined to produce the left- and right-travelling waves. The time-derivative *∂v*/*∂t* can be approximated by a difference operator, however in order to accommodate for the unknown propagation velocity and delay line length, common-mode leakage into *V_x_*, and losses in the delay line, we instead use a first-order least-mean-squares (LMS) adaptive filter^28^ for initial calibration. A signal source is applied to one port and the other is terminated; this produces a right-travelling wave on the line, but none travelling to the left. The left-travelling output *V*_−_ is used as an error signal for the LMS filter, suppressing any contribution from the right-travelling wave.

The real part of the reflection coefficient, seen looking out of the right port, is computed by a cross-correlation between left- and right-travelling waves. When this falls below 0.01, calibration is declared complete and filter updates cease. After calibration, we validate the system by configuring it as a reflectometer. Open and shorted measurements are made, yielding reflection coefficients of +1 and −1 respectively. The reflection coefficients of several resistors are also measured, again yielding the expected values.

We have used this device to implement the attack described above, using resistances *R_l_* = 1 kΩ, *R_h_* = 10 kΩ, and a coaxial transmission line of characteristic impedance *Z*_0_ = 50 Ω. The voltage sources are produced by an arbitrary waveform generator, producing independent normally-distributed voltages over a frequency range of 500 Hz–5500 Hz. The bandwidth *B* = 5 kHz results in an approximate correlation time of *B*^−1^ = 200 μs^29^. Each configuration is set and the covariance matrices from Eqn. 9 are measured during the setup phase. Resistor configurations are randomly selected for each test as would be the case in an operational system—though we used a pseudo-random number generator rather than a truly-random number generator—and the log-likelihood ratios are computed for the measured values of *v*_+_ and *v*_−_. Their differences are thresholded to compute (13).

## Author Contributions

L.J.G., A.A. and D.A. designed the study; L.J.G. wrote the paper; L.J.G. performed experiments and analyzed the data; A.A. and D.A. supervised the study; L.J.G., A.A. and D.A. discussed and interpreted the results; L.J.G., A.A. and D.A. proofed the paper.

## Supplementary Material

Supplementary Information Supplementary Note S1
