# Supplementary material for: A directional wave measurement attack against the Kish key distribution system
Source: Sci Rep. 2014 Sep 24;4:6461. doi: 10.1038/srep06461 (PMC4173037; doi:10.1038/srep06461)
Supplement: Supplementary Information — Supplementary Note S1 [file srep06461-s1.pdf]

A directional wave measurement attack against the Kish key distribution system Supplementary note S1

Lachlan J. Gunn,* Andrew Allison,† and Derek Abbott‡
School of Electrical and Electronic Engineering, The University of Adelaide, SA 5005, Australia

In this supplementary note we calculate the phase delay between the two ends of a terminated transmission line, as is carried out for the lumped RL model in [1, 2].

Let us consider the circuit of Figure 1, with $V_b = 0$. Then, in phasor form,

$$V_+ = V_a' + V_- \Gamma_a e^{-j2\pi f\tau} \quad (1)$$
$$= V_a' + V_+ \Gamma_a \Gamma_b e^{-j4\pi f\tau} \quad (2)$$
$$= \frac{V_a'}{1 - \Gamma_a \Gamma_b e^{-j4\pi f\tau}} \quad (3)$$
$$V_- = V_+ \Gamma_b e^{-j2\pi f\tau} \quad (4)$$
$$= \frac{V_a' \Gamma_b e^{-j2\pi f\tau}}{1 - \Gamma_a \Gamma_b e^{-j4\pi f\tau}}. \quad (5)$$
$$(6)$$

Then, the voltages V_1 and V_2 at the left- and right-most ends of the transmission line are, respectively,

$$V_1 = V_+ + V_- e^{-j2\pi f\tau} \quad (7)$$
$$= V_a' \frac{1 + \Gamma_b e^{-j2\pi f\tau}}{1 - \Gamma_a \Gamma_b e^{-j4\pi f\tau}} \quad (8)$$
$$V_2 = V_+ e^{-j2\pi f\tau} + V_- \quad (9)$$
$$= V_a' e^{-j2\pi f\tau} \frac{1 + \Gamma_b}{1 - \Gamma_a \Gamma_b e^{-j4\pi f\tau}}. \quad (10)$$

Thus,

$$\frac{V_2}{V_1} = e^{-j2\pi f\tau} \frac{1 + \Gamma_b}{1 + \Gamma_b e^{-j4\pi f\tau}}. \quad (11)$$
$$(12)$$

Noting that Γ_a and Γ_b are real in the KKD system, we may compute the phase difference ϕ_{21} between the two ends of the line as

$$-\phi_{21} = 2\pi f\tau + \angle(1 + \Gamma_b e^{-j4\pi f\tau}). \quad (13)$$

Then,

$$-\phi_{21} = 2\pi f\tau + \arctan\left(\frac{\Gamma_b \sin(-4\pi f\tau)}{1 + \Gamma_b \cos(-4\pi f\tau)}\right). \quad (14)$$

For a short line, $\tau \approx 0$ and so $\sin x \approx x$, $\cos x \approx 1$, and $\tan x \approx x$, reducing this to

$$-\phi_{21} \approx 2\pi f\tau \left(1 - 2\frac{\Gamma_b}{1 + \Gamma_b}\right). \quad (15)$$

We may write this quantity in terms of the resistance R_b, the length d of the line, and the capacitance C and inductance L per unit length using the formulae for Γ, Z_0, and ν from [3]:

$$-\phi_{21} \approx 2\pi f d\sqrt{LC}\left(1 - \frac{\sqrt{L/C}}{R_b}\right) \quad (16)$$
$$= 2\pi f \left(\tau - \frac{Ld}{R_b}\right). \quad (17)$$

This allows us to write the phase delay, as in [1], as

$$\tau_{\text{phase}} \approx \frac{Ld}{R_b} - \tau. \quad (18)$$

We have thus demonstrated that the d'Alembertian transmission line model, commonly used within electrical engineering [3, 4], replicates the results of [1]—except for an additional propagation delay—without resort to superluminal propagation.

[1] Chen, H.-P., Kish, L. B., Granqvist, C.-G. & Schmera, G. Do electromagnetic waves exist in a short cable at low frequencies? what does physics say? *Fluctuation and Noise Letters* **13**, 1450016 (2014).

[2] Chen, H.-P., Kish, L. B., Granqvist, C. G. & Schmera, G. On the "cracking" scheme in the paper "A directional coupler attack against the Kish key distribution system" by Gunn, Allison, and Abbott. *Metrology and Measurement Systems* (2014). In press, arXiv:1405.2034.

[3] Pozar, D. M. *Microwave Engineering* (Wiley, 1998).

[4] Coleman, C. *An Introduction to Radio Frequency Engineering* (Cambridge University Press, 2004).

* lachlan.gunn@adelaide.edu.au

† andrew.allison@adelaide.edu.au

‡ derek.abbott@adelaide.edu.au
